# Effects of feeding expeller-extruded high oleic soybeans in broiler diets on growth performance, blood profile, and meat quality

**DOI:** 10.1016/j.psj.2025.104960

**Published:** 2025-02-28

**Authors:** Ashir F. Atoo, Jorge Y. Perez-Palencia, Crystal L. Levesque, Keith Underwood, Kim Koch, Shane Mueller, Hari B. Krishnan, Jinsu Hong

**Affiliations:** a Department of Animal Science, South Dakota State University, Brookings, SD 57007, USA; b Northern Crops Institutes, Fargo, ND 58105, USA; c USDA-Agricultural Research Service, Plant Genetics Research Unit, Columbia, MO 65211, USA; d Department of Animal Science, University of Minnesota, Saint-Paul, MN 55108, USA

**Keywords:** Broiler, Immune health, Meat fatty acid composition, Oleic acid, Soybean

## Abstract

This study evaluated the effects of expeller-extruded high oleic soybeans (*TruSoya*) on growth performance, blood immune and antioxidant status, and meat quality of broilers compared with conventional soybean meal **(SBM**) and expeller-extruded conventional soybeans. A total of 288 one-day-old male broiler chicks (Cobb500; initial BW: 37.2 g) were allotted to one of 3 dietary treatments: 1) a corn basal diet with conventional SBM (**CSBM**); 2) a corn basal diet with expeller-extruded conventional soybeans (**EECS**); 3) a corn basal diet with expeller-extruded high oleic soybeans (**EEHS**) in a randomized complete block design. Diets were fed over 3 phases (starter phase 1: 0-1 weeks, grower phase 2: 1-3 weeks, and finisher phase 3: 3-6 weeks). At the end of each feeding phase, BW and feed consumption were measured to calculate average daily gain (**ADG**) and feed conversion ratio (**FCR**). At the end of the feeding trial (d 42), one bird per pen was bled for blood profile analysis, and two birds per pen were euthanized for breast and thigh collection. Data were subjected to analysis of variance testing the main effect of diet with pen as the experimental unit. Broilers fed the CSBM diet had greater (*P* < 0.05) BW at the end of each phase and overall ADG than those fed EECS and EEHS. Broilers fed EEHS showed greater (*P* < 0.05) final BW and overall ADG than EECS by 10.6 % and 10.7 %, respectively. The EEHS significantly increased (*P* < 0.05) blood basophil proportion compared with CSBM (15.7 % vs. 7.6 %). However, dietary treatment did not affect other blood cell count parameters and blood antioxidant status. Oleic acid contents for breast and thigh meats from broilers fed EEHS were higher (*P* < 0.05) than those of broilers fed CSBM and EECS by 63 % and 53 %, respectively. In conclusion, EEHS improved meat fatty acid composition with higher oleic acid contents and improved growth performance compared with EECS, suggesting its potential as a protein source for high quality broiler meat production.

## Introduction

According to the OECD-FAO's Agricultural Outlook report (OECD/FAO, 2020), consumption of poultry meat is projected to increase globally to 145 million tonnes by 2029 and it is expected to account for half of additional meat consumption. Increasing the concentration of oleic acid, which is the most common mono-unsaturated fatty acid (MUFA) in human diets, has been reported to be beneficial in reducing the risk of coronary heart diseases by 20-40 % and also valuable for weight management, and the prevention and management of obesity (Kris-Etherton, 1999; Liu et al., 2016). For humans, the easiest way to increase MUFA intake is by changing the fatty acid profile of foods they consume. Because monogastric animals, like broilers and pigs, deposit the same type of fatty acids they eat, the proportion of these “good” fatty acids can be increased in chicken meat by including feedstuffs with higher levels of these same “good” fatty acids in the poultry diet (Martins et al., 2015; Dal Bosco et al., 2022).

High oleic soybean is a non-GMO soybean with a high proportion of oleic acid (> 70 %) and linolenic fatty acids (>8 %) (Clemente and Cahoon, 2009). The high oleic soybean has excellent potential as an organic protein ingredient in the diets for organic and alternative poultry production systems. With respect to the fatty acid profile of the high oleic soybeans, expeller-extruded soybeans have a greater value as a feedstuff over conventional solvent extracted soybean meal (**SBM**) form to enhance the fatty acid profile of poultry meat for human consumption.

Dietary oleic acid or feedstuffs containing high oleic acid can enhance cellular antioxidant defense against mitochondrial oxidative stress (Duval et al., 2002; Rebolé et al., 2006). Dietary antioxidants regulate the expression of antioxidant enzymes and proteins to protect cells against oxidative damage triggered by injury and inflammation in the animal body (Surai and Kochish, 2019; Kikusato, 2021). Thus, supplementation of high oleic soybeans, which contain high concentration of oleic acid, in the poultry diet not only have benefits to human health via meat fatty acid profiles but also can enhance the health of the birds by improving antioxidant activity, leading to improved growth performance and low mortality, particularly under the antibiotic-free production system. Taken together, feeding expeller-extruded high oleic soybeans with higher fat content offers potential benefits, including enhanced meat fatty acid composition for human consumption and improved poultry health. However, expeller-extruded full-fat soybeans may have higher trypsin inhibitor activity compared to conventional solvent-extracted SBM, potentially compromising broiler growth performance.

There is limited information in the literature addressing these effects of incorporating high oleic soybeans in broiler diets. We hypothesized that dietary inclusion of expeller-extruded high oleic soybeans would result in greater improvements in broiler growth performance and health compared to expeller-extruded conventional soybeans. In addition, expeller-extruded high oleic soybeans would increase the proportion of “good” fatty acids in chicken meat compared to conventional solvent-extracted SBM. Thus, the objective of this study was to investigate the effects of expeller-extruded high oleic soybeans on the growth performance, blood immune and antioxidant status, and meat quality of broiler chickens compared with conventional SBM (solvent-extracted SBM) and expeller-extruded conventional soybeans.

## Materials and methods

The experimental animal procedures were reviewed and approved by the Institutional Animal Care and Use Committee at South Dakota State University, Brookings, SD, USA (SDSU-IACUC #2308-068E).

### Birds and housing

A total of 288 one-day-old Cobb500 male broiler chicks [average body weight (**BW)**: 37.2 g] were weighed and distributed to 36 floor pens (0.8 ⅹ 0.9 m^2^) with pine wood shavings (8 birds per pen based on BW). The pens were equipped with a heat lamp, one drinker, and one feeder, providing *ad libitum* access to water and feed during the entire period. The room temperature and lighting program followed the breeder recommendations. Brooder temperature was maintained at 34 ± 1°C for the first week. Room temperature was maintained at 31 ± 1°C for the first two weeks, 27 ± 1°C for the third week, 24 ± 1°C for the fourth week, 21 ± 1°C for the fifth week, and 18 ± 1°C for the sixth week. The general procedures for housing the birds followed the Ag Guide recommendation (Ag Guide, 2020).

### Experimental design

Three experimental diets, including a corn-based basal diet with conventional SBM (**CSBM**), a basal diet with expeller-extruded conventional soybeans (**EECS**), and expeller-extruded high oleic soybeans (**EEHS**), were allotted to the 36 pens in a randomized complete block design (*n* = 12 blocks, each comprised of 3 pens, with each pen representing a different treatment). The conventional SBM was solvent-extracted SBM sourced from Ag Processing Incorporated (Dawson, MN, USA). The Northern Crops Institute (NCI; Fargo, ND, USA) sourced conventional soybeans and high oleic soybeans (*TruSoya,* MN Soybean Research and Promotion Council, Mankato, MN, US) from Brushvale Seeds (Breckenridge, MN, USA). They processed the beans using an expeller-extruder (Instapro Jr Model 600, Insta-Pro International, Grimes, IA, USA) with 2.27 kg/min production rate at 152 ± 1°C. The analyzed composition of the SBM sources used in the current study is presented in Table 1. The diets were formulated to meet and exceed the nutrient requirement of the broilers based on Cobb500 Broiler Performance and Nutrition Supplement recommendation (Cobb-Vantress Inc., 2022) and provided in mash form. The diets were provided in a 3 phase-feeding scheme of starter phase: 0 to 1 weeks, grower phase: 1 to 3 weeks, and finisher phase: 3 to 6 weeks. All diets were formulated with the same energy and limiting amino acids (Lys, Met+Cys, and Thr) for each feeding phase (Table 2), and the analyzed composition of the diets is presented in Table 3.

**Table 1:** Analyzed nutrient composition of soybean products (As-fed basis).

Item	Conventional SBM	Expeller-Extruded conventional soybeans	Expeller-Extruded high oleic soybeans
Dry matter, %	89.29	95.24	95.55
Crude protein, %	43.13	36.12	39.68
Ether extract, %	1.56	19.88	19.09
Crude ash, %	5.37	4.71	4.79
Crude fiber, %	3.33	4.81	4.38
Neutral detergent fiber, %	7.08	7.92	7.75
Acid detergent fiber, %	5.01	6.21	6.46
Essential amino acids, %			
Arg	3.25	2.75	3.10
His	1.17	1.01	1.09
Ile	2.16	1.86	2.06
Leu	3.43	2.93	3.24
Lys	2.87	2.51	2.69
Met	0.62	0.52	0.53
Phe	2.29	1.97	2.23
Thr	1.70	1.46	1.58
Trp	0.71	0.62	0.67
Val	2.23	1.92	2.12
Nonessential amino acids, %			
Ala	1.91	1.62	1.76
Asp	4.98	4.25	4.68
Cys	0.69	0.57	0.63
Glu	8.10	6.62	7.33
Gly	1.87	1.62	1.76
Pro	2.22	1.87	2.09
Ser	1.90	1.67	1.84
Tyr	1.63	1.44	1.59
Fatty acid profile, %			
Palmitic acid (16:0)	14.97	10.77	7.19
Stearic acid (18:0)	4.00	4.13	3.92
Oleic acid (9c-18:1)	14.24	18.38	72.34
Linoleic acid (18:2n6)	52.07	52.94	7.85
Linolenic acid (18:3n3)	7.75	9.53	6.10
SFA^1^	20.15	15.93	12.33
MUFA^2^	16.65	20.63	72.92
PUFA^3^	59.82	62.53	14.01
PUFA:SFA	2.97	3.93	1.14
Trypsin inhibitor activity, TIU/mg	5.11	6.18	7.21

1 Saturated fatty acid.

2 Monounsaturated fatty acids.

3 Polyunsaturated fatty acids.

**Table 2:** Ingredient composition and nutrient composition of experimental diets.

Item	Starter		Grower		Finisher	
	Conventional SBM	Expeller-extruded soybeans	Conventional SBM	Expeller-extruded soybeans	Conventional SBM	Expeller-extruded soybeans
Ingredient, %						
Corn	57.48	57.86	60.54	59.63	61.40	60.51
Soybean meal	34.50	0.00	31.00	0.00	30.00	0.00
Extruded soybeans	0.00	36.50	0.00	34.00	0.00	33.00
Soybean oil	3.27	0.85	4.00	1.98	4.67	2.68
L-lysine·HCl	0.21	0.26	0.20	0.21	0.10	0.11
DL-methionine	0.32	0.32	0.29	0.28	0.25	0.24
L-threonine	0.17	0.16	0.11	0.09	0.06	0.03
L-valine	0.00	0.05	0.02	0.04	0.00	0.01
Limestone	1.40	1.40	1.32	1.32	1.20	1.20
Monocalcium phosphate 21 %	1.65	1.60	1.52	1.45	1.32	1.22
Sodium bicarbonate	0.10	0.10	0.10	0.10	0.10	0.10
Copper sulfate 25.2 %	0.05	0.05	0.05	0.05	0.05	0.05
Choline chloride 60 %	0.05	0.05	0.05	0.05	0.05	0.05
Salt	0.30	0.30	0.30	0.30	0.30	0.30
Broiler vitamin premix^1^	0.25	0.25	0.25	0.25	0.25	0.25
Broiler mineral premix^2^	0.25	0.25	0.25	0.25	0.25	0.25
Nutrient composition						
ME, Kcal/kg	3075	3075	3150	3150	3200	3200
Crude protein, %	21.07	21.11	19.69	20.15	19.30	19.78
Digestible Lys, %	1.22	1.22	1.12	1.12	1.02	1.02
Digestible Met+Cys, %	0.91	0.91	0.85	0.85	0.80	0.80
Digestible Thr	0.83	0.83	0.73	0.73	0.66	0.66
Digestible Val	0.90	0.90	0.85	0.85	0.82	0.80
Calcium, %	0.90	0.90	0.84	0.84	0.76	0.76
ATTD P, %	0.45	0.45	0.42	0.42	0.38	0.38

1 Provided the following per kilogram of diet: 10,000 IU vitamin A, 5,000 IU vitamin D_3_, 80 IU vitamin E, 3 mg thiamine, 9 mg riboflavin, 60 mg nicotinic acid, 15 mg pantothenic acid, 2 mg folic acid, 4 mg pyridoxine, and 0.15 mg biotin.

2 Provided the following per kilogram of diet: 100 mg manganese, 40 mg iron, 15 mg copper, 100 mg zinc, 1 mg iodine, and 0.35 mg selenium.

**Table 3:** Analyzed composition of experimental diets.^1^

Item	Starter			Grower			Finisher		
	CSBM	EECS	EEHS	CSBM	EECS	EEHS	CSBM	EECS	EEHS
Analyzed value, %									
Moisture	11.12	9.21	8.96	10.10	8.76	8.52	9.54	8.73	8.59
Crude protein	20.39	20.29	20.24	19.40	18.26	18.04	19.12	17.85	17.90
Crude fat	4.28	7.50	9.48	5.16	6.87	9.95	5.20	7.02	10.71
Ash	5.77	5.15	5.34	5.47	5.41	5.17	5.12	5.00	4.65
Crude fiber	2.13	2.82	2.64	2.18	2.56	2.49	2.18	2.52	2.41
NDF^2^	7.09	7.50	7.68	7.30	7.87	8.01	7.36	7.69	7.79
ADF^3^	3.88	4.08	4.24	3.57	3.82	4.09	3.73	3.70	3.98
Fatty acid profile, %									
Palmitic (16:0)	14.25	12.15	9.45	13.27	12.73	9.71	13.55	12.73	10.23
Stearic (18:0)	4.18	3.74	3.53	3.96	3.76	3.61	3.83	3.74	3.69
Oleic (9c-18:1)	25.74	19.36	57.95	22.57	19.64	50.32	24.14	19.70	45.30
Linoleic (18:2n6)	45.21	53.02	21.00	50.22	52.56	26.06	48.63	52.59	30.29
Linolenic (18:3n3)	4.65	7.30	4.94	5.14	7.06	5.15	5.43	7.14	5.57
SFA^4^	19.59	16.93	14.12	18.30	17.48	14.37	18.36	17.46	15.04
MUFA^5^	28.17	21.57	58.64	24.84	21.88	53.42	26.38	21.91	48.23
PUFA^6^	49.98	60.39	26.05	55.43	59.67	31.29	54.10	59.77	35.92
PUFA:SFA	2.55	3.57	1.84	3.03	3.41	2.18	2.95	3.42	2.39

1 CSBM: a corn-based basal diet with conventional SBM, EECS: a basal diet with expeller-extruded conventional soybeans, EEHS: a basal diet with expeller-extruded high oleic soybeans.

2 NDF: neutral detergent fiber.

3 ADF: acid detergent fiber.

4 SFA: saturated fatty acids.

5 MUFA: monounsaturated fatty acids.

6 PUFA: polyunsaturated fatty acids.

### Growth performance

The birds and feed were weighed at the end of each feeding phase to determine average daily gain (**ADG**), average daily feed intake (**ADFI**), and feed conversion ratio (**FCR**). Mortality was recorded daily and ADG and ADFI were corrected for mortality.

### Blood profiles

On day 42, one bird close to the average BW in each pen (12 birds per treatment) was selected and blood collected via brachial wing vein into a 5 ml plasma collection tube (BD Vacutainer®, K_2_E EDTA, Plymouth, UK) and a 5 ml serum collection tube (BD Vacutainer®, Plymouth, UK). The collected blood samples in the serum tubes were centrifuged at 1,872 x g for 20 min at 4 °C, after then the serum samples were transferred into new microtubes and stored at - 20 °C for later determination of glutathione peroxidase (**GPx**), superoxide dismutase (**SOD**), protein carbonyl (**PC**) concentrations to assess antioxidant status of the broilers. The GPx, SOD, and PC analyses were determined by the enzyme-linked immunosorbent assay (**ELISA**) using commercially available kits (GPx: Chicken Glutathione Peroxidase 8 ELISA Kit, Biomatik USA, LLC, Wilmington, DE, USA; SOD: Chicken Superoxide Dismutases ELISA Kit, MyBioSource Inc., San Diego, CA, USA; protein carbonyl: Chicken Protein Carbonyl ELISA Kit, MyBioSource Inc., San Diego, CA, USA). The collected plasma samples in the EDTA-coated tubes were immediately sent to the Animal Disease Research and Diagnostic Laboratory, South Dakota State University (Brookings, SD, USA) for determination of complete blood count (**CBC**) by using a chemistry analyzer (Vet Axcel®, Alfa Wassermann Inc., NJ, USA).

### Meat quality

On day 42, two birds from each pen, with a BW close to the pen's average BW, were selected and euthanized via CO_2_ asphyxiation (one bird was the same bird used for blood collection). Breast and thigh meats collected from one bird were used for analysis of meat color, drip loss, and cook loss. Breast and thigh meats collected from the other bird were used for analysis of fatty acid profile. Analyses for meat color, drip loss, and cook loss were performed by SDSU Meat Lab (Brookings, SD, USA). The L*, a*, and b* values were measured by using a handheld colorimeter (Chroma Meta CR-410, Konica Minolta, Ramsey, NJ, USA) equipped with a 50 mm aperture, 0° viewing angle, 2° standard observer, pulsed xenon lamp light source, and calibrated with a white tile (L* = 97.38, a* = 0.06, b* = 1.82).

Drip loss was determined by weighing each breast and thigh and suspending each breast or thigh within a gallon-size plastic bag. Breasts and thighs were allowed to drip at 4°C for 24h. Each breast or thigh was weighed again after 24 h and drip loss was calculated as a percentage loss of initial weight. Broiler breasts and boneless thighs designated for cooking loss were thawed for 24 h at 4.4°C. Prior to cooking, all breasts and thighs were weighed, and the initial weight was recorded for cooking loss. All cuts were cooked to a target internal temperature of 74°C using an electric clamshell grill (George Foreman, Model GR2144P, Middleton, WI, USA). Peak internal temperatures were recorded for each cut using a digital thermocouple (Atkins Aqua Tuff NSF Series, Cooper-Atkins Corporation, Middlefield, CT, USA). Cooked breasts and thighs were allowed to cool for 4 h at room temperature. Breasts and thighs were weighed again to determine cooking loss.

The fatty acid composition of chicken breast and thigh meat, including saturated fatty acids (**SFA**), monounsaturated fatty acids (**MUFA**), and polyunsaturated fatty acids (**PUFA**), were analyzed by gas chromatography–mass spectrometry by a commercial analytical laboratory (FoodChain ID, Chantilly, VA, USA).

### Ingredient and feed analysis

The test soybean products and experimental diets were ground finely to pass through a 0.75-mm screen using a centrifugal mill (model Zm200; Retsh GmbH, Haan, Germany). Test feedstuffs and diets were analyzed for dry matter (**DM**), crude protein (**CP**), ether extract (**EE**), crude ash, and fiber characteristics including crude fiber, acid detergent fiber (**ADF**), neutral detergent fiber (**NDF**), amino acids (**AA**), fatty acids (**FA**), and trypsin inhibitor activity.

The samples were analyzed for DM by oven drying at 135°C for 2 h (method 930.15), CP by a combustion procedure (method 990.03), EE (method 2003.06), crude ash (method 942.05), and crude fiber (method 978.10) as per AOAC (2007). The samples were analyzed for ADF and NDF as described by Van Soest et al. (1991) on an Ankom 200 Fiber Analyzer (Ankom Technology, Fairport, NY). The samples were analyzed for AA (method 982.30 E) and FA (method 996.06 and 965.49) at the University of Missouri Experiment Station laboratories (Columbia, MO, USA) using the AOAC (2007). Trypsin inhibitor activity was analyzed using a recent simplified method proposed by USDA-ARS (Columbia, MO, USA; Kim and Krishnan, 2023).

### Statistical Analysis

The UNIVARIATE procedure of SAS (SAS Inst., Inc., Cary, NC, USA) was used to confirm the homogeneity of variance and to analyze for outliers. Data were subjected to analysis of variance using the MIXED procedure of SAS with the pen as the experimental unit. The model included the dietary treatment as a fixed effect and the pen block as a random effect. Treatment means were separated by the probability of difference using Tukey's adjustment where *P* < 0.05 was considered significant. If pertinent, tendency (0.05 ≤ *P* < 0.10) was also reported.

## Results

The analyzed nutrient composition of conventional SBM and expeller-extruded soybeans are presented in Table 1. The CP value for conventional SBM was greater than expeller-extruded conventional soybeans and expeller-extruded high oleic soybeans by 19.4 % and 8.6 %, respectively, whereas EE content for expeller-extruded soybeans was greater than conventional SBM. The expeller-extruded high oleic soybeans contained higher oleic acid composition (72.3 %) than conventional SBM (14.2 %) and expeller-extruded conventional soybeans (18.4 %). Trypsin inhibitor activity for expeller-extruded conventional soybeans and high oleic soybeans were greater than conventional SBM by 1.07 and 2.10 units (TIU/mg), respectively.

Overall, bird health was good throughout the entire experimental period, with no visible signs of disorders, illness, or disease as diagnosed by the university veterinarian. The overall livability was high (96.5 %), with no treatment-related effects on mortality; there were 4, 2, and 4 mortalities for CSBM, EECS, and EEHS, respectively, during the entire period. The effect of expeller-extruded high oleic soybeans inclusion in broiler diets on growth performance is presented in Table 4. Broilers fed CSBM showed greater (*P* < 0.05) BW at 1, 3, and 6 weeks than broilers fed EECS. Also, broilers fed CSBM diets showed greater (*P* < 0.05) ADG for starter phase and overall period and ADFI for stater and grower phases and overall period than those of broilers fed EECS and EEHS. Broilers fed EEHS showed greater (*P* < 0.05) BW and ADG for each phase and overall period and ADFI for grower and finisher phases and overall period than those fed EECS. However, dietary treatment did not affect the overall FCR of broilers. Although not statistically significant, there were improvements of 8.6 % and 9.4 % in FCR during the starter phase and 7.7 % and 5.6 % in FCR during the grower phase in broilers fed EECS and EEHS, respectively, compared to those fed CSBM.

**Table 4:** Effect of extruded high oleic soybeans on the growth performance of broiler chickens.

Item	Treatment^1^			SEM^2^	*P*-value
	CSBM	EECS	EEHS		
Body weight, g/bird					
Initial	37	37	37	0.3	0.994
Week 1	202^a^	171^c^	184^b^	3.2	<.001
Week 3	955^a^	820^c^	913^b^	14.0	<.001
Week 6	3069^a^	2674^c^	2958^b^	38.2	<.001
Average daily gain, g/d					
0 - 1 weeks	24^a^	19^c^	21^b^	0.5	<.001
1 - 3 weeks	54^a^	46^b^	52^a^	1.0	<.001
3 - 6 weeks	101^a^	88^b^	97^a^	1.6	<.001
0 - 6 weeks	72^a^	63^c^	70^b^	0.9	<.001
Average daily feed intake, g/d					
0 - 1 weeks	30^a^	22^b^	24^b^	0.8	<.001
1 - 3 weeks	77^a^	61^c^	70^b^	2.1	<.001
3 - 6 weeks	163^a^	145^b^	156^a^	2.2	<.001
0 - 6 weeks	112^a^	96^c^	106^b^	1.5	<.001
Feed conversion ratio					
0 - 1 weeks	1.28	1.17	1.16	0.041	0.101
1 - 3 weeks	1.43	1.32	1.35	0.042	0.156
3 - 6 weeks	1.62	1.64	1.61	0.022	0.578
0 - 6 weeks	1.55	1.54	1.52	0.019	0.544

Means correspond to average of 12 pens per treatment.

^abc^Within a row, means without a common superscript differ (*P* < 0.05).

1 CSBM: a corn-based basal diet with conventional SBM, EECS: a basal diet with expeller-extruded conventional soybeans, EEHS: a basal diet with expeller-extruded high oleic soybeans.

2 Standard error of the mean.

Effects of expeller-extruded high oleic soybeans on the blood profile of broilers has been presented in Table 5. Dietary treatment did not affect the CBC result, including white blood cell count, red blood cell count, packed cell volume (**PCV**), lymphocytes, eosinophils, and concentrations of hemoglobin, mean corpuscular hemoglobin concentration (**MCHC**), and total protein. However, broilers fed EEHS had greater (*P* < 0.05) blood basophil proportion compared with that of broilers fed CSBM. Dietary treatment did not affect the serum concentrations of antioxidant markers GPx, SOD, and PC in broilers on day 42.

**Table 5:** Effect of extruded high oleic soybeans on the blood profile of broiler chickens.

Item^1^	Treatment^2^			SEM^3^	*P*-value
	CSBM	EECS	EEHS		
WBC, 10^−3^/μL	11.77	10.82	7.77	2.920	0.606
RBC, 10^−6^/μL	1.89	1.88	1.72	0.060	0.134
PCV, %	34.20	33.67	31.17	1.148	0.170
HGB, g/dL	9.60	9.00	10.00	0.630	0.587
MCHC, g/dL	28.14	26.72	32.18	1.808	0.143
Total protein, g/dL	2.54	2.23	2.20	0.230	0.477
Hets/Neuts %	49.00	47.17	44.50	3.087	0.576
Lymphocytes %	36.40	35.33	33.83	2.547	0.765
Basophils %	7.60^b^	11.67^ab^	15.67^a^	1.935	0.022
Eosinophils %	7.00	5.83	6.00	1.404	0.793
GPx, U/mL	171.2	160.0	122.3	18.42	0.160
SOD, U/mL	22.94	16.25	15.99	2.594	0.116
PC, ng/mL	2.72	1.54	2.16	0.428	0.166

Means correspond to 12 blood samples per treatment, 1 broiler per replicate pen, selected close to the average pen body weight at 42 d.

^ab^Within a row, means without a common superscript differ (*P* < 0.05).

1 WBC: white blood cell, RBC: red blood cell, PCV: packed cell volume, HGB: hemoglobin, MCHC: Mean corpuscular hemoglobin concentration, Hets/Neuts: heterophil extracellular traps/neutrophils, GPx: glutathione peroxidase, SOD: Superoxide dismutase, PC: protein carbonyl.

2 CSBM: a corn-based basal diet with conventional SBM, EECS: a basal diet with expeller-extruded conventional soybeans, EEHS: a basal diet with expeller-extruded high oleic soybeans.

3 Standard error of the mean.

Effects of extruded high oleic soybeans on meat quality of broiler chickens has been presented in Table 6. Although dietary treatment did not affect the meat color of chicken breast, EECS treatment tended to increase (*P* = 0.085) drip loss of chicken breast. The redness (a*) of meat color from birds fed EECS was lower (*P* < 0.05) than CSBM and EEHS, whereas dietary treatment did not affect the lightness (L), yellowness (b), drip loss, and cooking loss of chicken thigh.

**Table 6:** Effect of extruded high oleic soybeans on the carcass characteristic of chicken breast and thigh meats.

Item	Treatment^1^			SEM^2^	*P*-value
	CSBM	EECS	EEHS		
**Breast meat**					
Meat color^3^					
L*	60.4	60.6	61.4	0.70	0.585
a*	15.1	14.8	14.6	0.39	0.681
b*	13.5	13.7	14.0	0.50	0.775
Drip loss, %	1.65^y^	2.21^x^	1.66^y^	0.198	0.085
Cooking loss	28.4	28.6	27.2	0.87	0.501
**Thigh meat**					
Meat color					
L*	57.1	58.4	58.1	0.86	0.578
a*	16.9^a^	15.3^b^	16.2^ab^	0.40	0.024
b*	10.7	10.8	10.9	0.66	0.900
Drip loss, %	0.21	0.24	0.20	0.412	0.824
Cooking loss, %	25.8	25.8	24.4	0.99	0.483

Means correspond to 12 breast samples and 12 thigh samples per treatment, 1 broiler per replicate pen, selected close to the average pen body weight at 42 d.

^ab^Within a row, means without a common superscript differ (*P* < 0.05).

^xy^Within a row, means without a common superscript differ (0.05 ≤ *P* < 0.10).

1 CSBM: a corn-based basal diet with conventional SBM, EECS: a basal diet with expeller-extruded conventional soybeans, EEHS: a basal diet with expeller-extruded high oleic soybeans.

2 Standard error of the mean.

3 meat color description: L* (brightness), a* (redness), b* (yellowness).

Effects of expeller-extruded high oleic soybeans on FA composition of chicken breast and thigh meats are presented in Table 7, Table 8, respectively. In breast meat, EEHS increased (*P* < 0.05) the proportion of MUFA, mainly oleic acid, and reduced (*P* < 0.05) the proportions of SFA (palmitic acid, stearic acid, and Behenic acid) and PUFA (linoleic acid, linolenic acid) compared with CSBM and EECS. In thigh meat, EEHS increased (*P* < 0.05) the proportion of MUFA, mainly oleic acid (18:1), and reduced (*P* < 0.05) the proportions of two SFA, including palmitic acid (16:0) and myristic acid (17:9), and PUFA, including linoleic acid (18:2), eicosatrienoic acid (20:3), and timnodonic acid (20:5), compared with CSBM.

**Table 7:** Effect of soybean sources on the fatty acids profile in breast meat of broilers.

Item	Treatment^1^			SEM^2^	*P*-value
	CSBM	EECS	EEHS		
Saturated fatty acids (SFA), %					
Myristic acid (C14:0)	0.38^a^	0.29^b^	0.27^b^	0.011	<.001
Pentadecanoic acid (C15:0)	0.06	0.06	0.06	0.003	0.078
Palmitic acid (C16:0)	19.97^a^	16.34^b^	14.87^c^	0.291	<.001
Margaric acid (C17:0)	0.13^b^	0.17^a^	0.13^b^	0.006	<.001
Stearic acid (C18:0)	5.96^a^	5.09^b^	4.02^c^	0.140	<.001
Arachidic acid (C20:0)	0.09	0.09	0.09	0.003	0.486
Behenic acid (C21:0)	0.20^a^	0.22^a^	0.10^b^	0.010	<.001
Monounsaturated fatty acids (MUFA), %					
Myristoleic acid (C14:1, cis)	0.09^a^	0.05^c^	0.07^b^	0.005	<.001
Palmitoleic acid (C16:1, cis)	3.71^a^	2.24^c^	2.92^b^	0.168	<.001
Oleic acid (C18:1, cis)	33.37^b^	29.23^c^	54.45^a^	0.484	<.001
Gondoic acid (C20:1)	0.27^b^	0.21^c^	0.33^a^	0.008	<.001
Erucic acid (C22:1)	0.12^ab^	0.24^a^	0.01^b^	0.054	0.017
Polyunsaturated fatty acids (PUFA), %					
Linoleic acid (C18:2, cis)	31.63^b^	39.89^a^	18.40^c^	0.646	<.001
α-Linolenic acid (C18:3, n3)	3.12^c^	4.86^a^	3.66^b^	0.124	<.001
Linolenic acid (C18:3, n6)	0.25^a^	0.24^a^	0.17^b^	0.009	<.001
Eicosatrienoic acid (C20:3, n3)	0.35	0.39	0.24	0.049	0.106
Timnodonic acid (C20:5, n3)	0.21^ab^	0.24^a^	0.18^b^	0.015	0.022
SFA, %	26.84^a^	22.31^b^	19.55^c^	0.370	<.001
MUFA, %	37.56^b^	31.97^c^	57.79^a^	0.594	<.001
PUFA, %	35.59^b^	45.72^a^	22.67^c^	0.755	<.001

Means correspond to 12 breast samples per treatment, 1 broiler per replicate pen, selected close to the average pen body weight at 42 d.

^abc^Within a row, means without a common superscript differ (*P* < 0.05).

1 CSBM: a corn-based basal diet with conventional SBM, EECS: a basal diet with expeller-extruded conventional soybeans, EEHS: a basal diet with expeller-extruded high oleic soybeans.

2 Standard error of the mean.

**Table 8:** Effect of soybean sources on the fatty acids profile in thigh meat of broilers.

Item	Treatment^1^			SEM^2^	*P*-value
	CSBM	EECS	EEHS		
Saturated fatty acids (SFA), %					
Myristic acid (C14:0)	0.38^a^	0.28^b^	0.28^b^	0.009	<.001
Pentadecanoic acid (C15:0)	0.06	0.06	0.06	0.002	0.284
Palmitic acid (C16:0)	19.08^a^	15.24^b^	14.71^b^	0.207	<.001
Margaric acid (C17:0)	0.12^b^	0.16^a^	0.13^b^	0.005	<.001
Stearic acid (C18:0)	5.16	4.51	8.39	2.797	0.573
Arachidic acid (C20:0)	0.08	0.09	0.12	0.014	0.177
Behenic acid (C21:0)	0.17	0.18	1.16	0.660	0.476
Monounsaturated fatty acids (MUFA), %					
Myristoleic acid (C14:1, cis)	0.10^a^	0.05^c^	0.07^b^	0.004	<.001
Palmitoleic acid (C16:1, cis)	4.16^a^	2.25^c^	3.02^b^	0.142	<.001
Oleic acid (C18:1, cis)	34.05^b^	28.66^b^	52.04^a^	1.883	<.001
Gondoic acid (C20:1)	0.26^b^	0.19^c^	0.31^a^	0.011	<.001
Polyunsaturated fatty acids (PUFA), %					
Linoleic acid (C18:2, cis)	32.46^b^	42.11^a^	16.69^c^	1.002	<.001
α -Linolenic acid (C18:3, n3)	3.25^b^	5.48^a^	3.37^b^	0.189	<.001
Linolenic acid (C18:3, n6)	0.25	0.26	0.46	0.188	0.652
Eicosatrienoic acid (C20:3, n3)	0.22^a^	0.24^a^	0.13^b^	0.017	0.003
Timnodonic acid (C20:5, n3)	0.18^b^	0.20^a^	0.12^c^	0.006	<.001
SFA, %	25.07	20.52	22.72	2.205	0.357
MUFA, %	38.57^b^	31.15^c^	55.56^a^	1.881	<.001
PUFA, %	36.36^b^	48.33^a^	20.56^c^	1.172	<.001

Means correspond to 12 thigh samples per treatment, 1 broiler per replicate pen, selected close to the average pen body weight at 42 d.

^abc^Within a row, means without a common superscript differ (*P* < 0.05).

1 CSBM: a corn-based basal diet with conventional SBM, EECS: a basal diet with expeller-extruded conventional soybeans, EEHS: a basal diet with expeller-extruded high oleic soybeans.

2 Standard error of the mean.

## Discussion

### Growth performance

The present study investigated the effects of EEHS in broiler diets on growth performance, blood immune and antioxidant markers, and meat quality, compared with CSBM and EECS. It was hypothesized that the inclusion of high oleic soybeans in the diet would result in improved growth performance and health outcomes, along with an enhanced fatty acid profile in the meat. The findings of this study support the hypothesis to some extent, as broilers fed EEHS exhibited increased oleic acid content in the meat and had a better growth performance compared to EECS, although growth performance was generally better in broilers fed CSBM. The results from this are further discussed below.

The relatively lower growth performance for the starter period in broilers fed the extruded soybeans diets could partly have been due to the higher trypsin inhibitor activity of EECS (2.04-2.26 TIU/mg; calculated values) and EEHS (2.38-2.63 TIU/mg; calculated values) compared with CSBM (1.53-1.76 TIU/mg; calculated values). Solvent-extracted SBM has an additional heating process which partially inactivates trypsin inhibitor during the toasting and solvent-extraction rather than the extrusion process which does not have the additional heating step (Heger et al., 2016). The observations in the current study agreed with the growth performance result of Ali et al. (2024a), where broilers fed the solvent-extracted SBM showed greater BW and ADG for 0 to 35 days than those of broilers fed diets with expeller-extruded conventional soybeans or expeller-extruded high oleic soybeans. However, they found no difference in growth performance between expeller-extruded diets. This finding contradicted the result of the current study, where broilers provided the expeller-extruded high-oleic soybean diet had greater final BW, ADG, and ADFI than broilers provided the expeller-extruded conventional soybeans. Although both studies formulated the relevant experimental diets with similar energy and protein levels, Ali et al. (2024a) included 25 % and 31 % of expeller-extruded soybeans (0-14d and 15-35d, respectively) in corn-based diet as partial replacement for solvent-extracted SBM (0-14d: 16.5 %, 15-35d: 6.9 %) whereas the current study included 36.5 to 33.0 % of expeller-extruded soybeans in corn-based diet as complete replacement of solvent-extracted SBM. The apparent metabolizable energy value for expeller-extruded high oleic-full fat soybeans was reported to be 23 kcal/kg (DM basis) greater than that for expeller-extruded conventional full fat soybeans in broilers by (Ali et al., 2024b). Thus, the greater performance in broilers fed EEHS compared with EECS could partly have been due to differences in metabolizable energy content between the expeller-extruded conventional soybeans and expeller-extruded high oleic soybeans.

Previous studies indicated that trypsin inhibitors could be considered a major antinutritional factor of soybean products due to their negative impacts on growth performance and nutrient digestibility in broilers (Clarke and Wiseman, 2005; Aderibigbe et al., 2020; Kuenz et al., 2022). Trypsin inhibitors can interfere with biological activities, including the protein digestion pathway and the exocrine function of the pancreas (enzyme secretion and endogenous loss), and thereby reduce the digestion of protein and other nutrients in poultry (Erdaw and Beyene, 2018). It should be noted that young broiler chicks are more susceptible to the trypsin inhibitor activity of dietary soybean products than older broilers (Clarke and Wiseman, 2005,^2007^; Erdaw et al., 2017). Thus, greater growth performance in CSBM compared with EECS and EEHS could partly have been due to the lower trypsin inhibitor activity for conventional SBM and CSBM diet than expeller-extruded soybeans, EECS, and EEHS

### Blood profile

Inclusion of high-oleic soybeans in broiler diets resulted in an increased proportion of blood basophil. Avian basophils play a critical role in early inflammatory and immediate hypersensitivity reactions, which degranulate to release histamine and other vasoactive amines during the inflammatory reaction (Maxwell and Robertson, 1995). Previous studies indicated that a significant increase in the number of basophils in poultry may result from prolonged stress affecting cell-mediated immunity (reviewed by Maxwell and Robertson, 1995). In the current study, the birds were housed in identical environmental conditions throughout the feeding trial, and there was no clinical issue observed by a veterinarian and investigators. Regarding the fact that soybean products contain antinutritional factors for poultry, such as trypsin inhibitors and allergenic proteins (glycinin and beta-conglycinin), the dietary antinutritional factors from extruded high-oleic soybean may have contributed to the immune response of the broilers (Woyengo et al., 2017). However, the similar or numerically improved FCR in broilers fed EEHS in the present study suggests that the elevated basophil level was not sufficient to indicate compromised health or immunity. Further investigations are needed to explore the potential implications of high-oleic soybean products on poultry health.

### Blood antioxidant markers

Dietary lipid sources may differentially influence antioxidant enzyme activity in poultry (Chen et al., 2012, 2015). For instance, Long et al. (2019) reported that increasing the inclusion level of palm oil, which primarily contains palmitic acid, oleic acid and linoleic acid (Sundram et al., 2003), linearly increased serum SOD levels; however, it did not affect serum GPx levels in broilers. Sierżant et al. (2023) reported no differences in blood total antioxidant status and GPx levels between a high oleic acid diet with rapeseed oil and a low oleic acid diet with flaxseed oil. Previous studies reported that dietary oleic acid could exhibit an antioxidant activity against oxidative stress in broilers (Duval et al., 2002; Rebolé et al., 2006). Regarding the results of the current study and previous broiler studies, the difference in fatty acid composition in the experimental diets was not sufficient to significantly affect the blood antioxidant activity of broiler chickens under the tested conditions. One possible explanation for these findings is that the birds were not subjected to oxidative stress at the time of sample collection, which may have limited the observable impact of oleic acid on antioxidant responses. Further studies incorporating oxidative stress challenges could help elucidate the role of dietary oleic acid in modulating antioxidant activity in broilers.

### Carcass traits

Meat color is an important consumer purchasing and consumption acceptance factor. Pale, soft, and exudative meat has been considered of poor consumer acceptance due to the pale color and appearance, increased cooking loss and drip loss (Johnston et al., 2005). A rapid conversion of glycogen to lactic acid in the meat causes a significant reduction in pH, resulting in pale, soft, and exudative meat (Fletcher et al., 2000; Garcia et al., 2010). Toomer et al. (2019) indicated that feeding broilers a high oleic diet from inclusion of peanuts did not affect drip loss and shear force of the breast meat, however, it tended to increase cook loss compared with a corn-SBM based diet. Also, they reported no differences in breast meat color (L*, a*, and b*) on postmortem day 1 between the corn-SBM based diet and the high oleic peanut diet. Ali et al. (2024a) observed that chicken breast from broilers fed expeller-extruded conventional full-fat soybeans had greater pH 24 h post-mortem than those from broilers fed expeller-extruded high oleic full-fat soybeans, however, no significant differences were found in meat color, drip loss, and cook loss of the breast meat. Although post-mortem pH changes in the meat was not measured in this work, the decrease in redness (a* value) of breast meat and the increase in drip loss of thigh meat for EECS may partly be due to the rapid decline in pH compared to the other treatments. Therefore, further study should explore the physiological and metabolic mechanisms underlying how dietary fatty acid sources or meat fatty acid composition affect the pH of chicken meat.

### Meat fatty acid profile

Dietary fatty acid composition has shown a significant correlation to meat fatty acid composition in poultry (Lopez-Ferrer et al., 1999; Tuunainen et al., 2016; Semwogerere et al., 2019). The fatty acid profile of chicken breast meat observed in the current study was in agreement with the result of Ali et al. (2024a), who fed expeller-extruded high oleic full-fat soybeans to broilers. They reported that broilers fed extruded high oleic full-fat soybeans showed higher oleic acid (18:1) and lower linoleic acid (18:2), palmitic acid (16:0) and stearic acid (18:0) in breast fillets than those of broilers fed conventional SBM or expeller-extruded conventional soybeans. Also, Slaughter et al. (2019) reported that broilers fed high oleic SBM or high oleic soybean oil had higher proportions of oleic acid (18:1) and MUFA with lower proportions of linoleic acid (18:2) and PUFA in breast and thigh meat compared with those of broilers fed corn-based diet with conventional soybean meal and oil. Toomer et al. (2020) found that feeding high oleic peanuts (80 % oleic acids and 2 % linoleic acid) to broilers for 42 days increased oleic acid and MUFA contents and decreased SFA, including palmitic acid and stearic acid in chicken breast. Altering the dietary fatty acid composition through the incorporation of high-oleic soybean meal in broiler diets resulted in a significant enhancement of oleic acid and MUFA content in both breast and thigh meats.

In conclusion, this study has shown that the inclusion of expeller-extruded high oleic soybeans in broiler diets influenced growth performance, blood profiles, and meat composition. Broilers fed CSBM had better growth performance than those fed expeller-extruded soybeans. However, broilers fed EEHS showed a numerical improvement of FCR compared with those fed CSBM and better growth performance than those fed EECS. While the differences in the fatty acid profile of EEHS were not enough to alter the antioxidant status of the broilers, it enhanced the oleic acid and MUFA contents in both the breast and thigh meat and reduced the content of SFA, particularly in the breast meat without adversely affecting the meat quality of the chicken breast and thigh.

## Declaration of competing interest

The authors declare the following financial interests/personal relationships which may be considered as potential competing interests:

Jinsu Hong reports financial support was provided by Minnesota Soybean Research and Promotion Council. If there are other authors, they declare that they have no known competing financial interests or personal relationships that could have appeared to influence the work reported in this paper.
